# Draft genome sequence of *Catenibacterium mistuoki* isolated from the fecal sample of a melanoma patient with complete response to immunotherapy

**DOI:** 10.1128/mra.00732-25

**Published:** 2025-11-06

**Authors:** Florencia Peñalba, Andrés Parada, Nabila Elgul, Maria Isabel Alonso, Gregorio Iraola, Nora Berois, Eduardo Osinaga, Nadia Riera

**Affiliations:** 1 Microbial Genomics Lab, Institut Pasteur de Montevideo 123939, Montevideo, Uruguay; 2 Center for Innovation in Epidemiological Surveillance, Institut Pasteur de Montevideo 123939, Montevideo, Uruguay; 3 Oncology Department, Centro Asistencial del Sindicato Médico del Uruguay 671480 , Montevideo, Uruguay; 4 Wellcome Sanger Institute 47665, Hinxton, United Kingdom; 5 Glycobiology and Tumor Immunology, Institut Pasteur de Montevideo, Montevideo, Uruguay; Nanchang University, Nanchang, Jiangxi, China

**Keywords:** immunotherapy, *Catenibactiruim*, anaerobes

## Abstract

A strain of *Catenibacterium* spp. was isolated from a fecal sample of a patient diagnosed with stage IV melanoma in December 2008. The patient began treatment with the immune checkpoint inhibitor pembrolizumab in May 2016 and has since exhibited a sustained complete response to the therapy.

## ANNOUNCEMENT

Catenibacterium is a Gram-positive, non-spore-forming, anaerobic genus belonging to the family *Erysipelotrichidae*. To date, only one species has been validly published under the International Code of Nomenclature of Prokaryotes: *Catenibacterium mistuoki*, which was first isolated from the feces of highland inhabitants of Papua New Guinea living at high altitudes (1). In addition, a subspecies, *Catenibacterium tridentinum*, has also been identified (2).

The *Catenibacterium mistuoki* strain was isolated in an anaerobic chamber (95% N_2_/5% H_2_) from the feces of a patient diagnosed with stage IV melanoma in Uruguay. The fresh sample was homogenized in PBS, serially diluted, and plated at 10−5 and 10^−6^ dilutions on a YCFA agar medium. After incubation at 37°C for 2 days, a single colony was inoculated in the YCFA liquid medium and incubated for an additional 24 h at 37°C. The YCFA medium was prepared in-house following the standard formulation (3).

Genomic DNA was extracted from the liquid culture using the PureLink DNA Purification Kit (Thermo Fisher Scientific, USA) following the manufacturer’s standard protocol. The quality and quantity of the extracted DNA were assessed using the Qubit dsDNA Quantification Assay Kit (Thermo Fisher Scientific, USA) prior to library preparation. Whole-genome sequencing (WGS) was performed using both Illumina and Oxford Nanopore sequencing technologies. Illumina sequencing was performed on the NovaSeq SP platform at the Wellcome Sanger Institute (Hinxton, United Kingdom). Libraries were prepared following the institute’s standard protocols for the NovaSeq SP platform without modifications. The sequencing yielded paired-end reads with a length of 151 bp, generating approximately 1.61 million paired-end reads (~210 Mbp) with a GC content of 33%. Read quality was assessed using FastQC v0.12.1 (4). According to FastQC analysis, the Illumina data set comprised approximately 1.39 million reads, totaling 210 Mbp, with a GC content of 33%. All bioinformatics tools were run using default parameters. Genome assembly was performed using SPAdes v3.15.5 (5), and the assembly quality was assessed with QUAST v5.2.0 (6). The final assembly had a total length of 2,248,535 base pairs and consisted of 114 contigs.

Oxford Nanopore sequencing was performed in Centro de Innovación en Vigilancia Epidemiológica (CiVE) at the Institut Pasteur de Montevideo. Genomic DNA was sheared using g-TUBEs (Covaris, USA). DNA was then fragmented by centrifugation at 6,000 RPM for 60 s, producing a fragment distribution around 10 kb. The DNA was used for library preparation with the Oxford Nanopore Technologies Native Barcoding Kit SQK-NBD114.24 following the manufacturer’s instructions. Sequencing was performed on a GridION with FLO-MIN106D flow cells. Basecalling was performed using the high-accuracy model v3.3 (450 bps) with a minimum Q score of 9. The software versions used were as follows: MinKNOW v24.11.8, Bream v8.2.5, Configuration v6.2.12, Dorado v7.6.7, and MinKNOW Core v6.2.6. Raw data were filtered using NanoFIlt v2.8.0 (7) with the following parameters: a minimum average quality score of 8, a minimum read length of 200 bp, and trimming of 70 bases from both the 5′ and 3′ ends of each read (-headcrop 70—tailcrop 70). After filtering, a total of 1,327 long reads remained, with a mean read length of 3,334 bp, a mean quality score of 11.3, and an ONT read N50 of 39,664 bp. Filtered Nanopore reads were aligned to the SPAdes v3.15.5 (5) Illumina-based assembly using Minimap2 v2.26 (8). The resulting SAM file was used directly as input for one round of polishing with Racon v1.4.20 (9). The polished hybrid assembly was then evaluated with QUAST v5.2.0 (Table 1) (6).

**TABLE 1:** Assembly statistics generated by QUAST v5.2.0 for the polished hybrid genome

Assembly	
Contigs (>= 0 bp)	50
Contigs (>1,000 bp)	50
Contigs (>5,000 bp)	42
Contigs (>10,000 bp)	38
Contigs (>25,000 bp)	30
Contigs (>50,000 bp)	18
Total length (>0 bp)	2134571
Total length (>1,000 bp)	2134571
Total length (>5,000 bp)	2134571

Taxonomic classification was performed using GTDB-Tk v2.4.1 with database version r226 (10). The genome was classified as belonging to the genus *Catenibacterium*, with *C. mitsoukai* as its closest placement reference (ANI 95.66 against GCA_964259225.1).

**Fig 1:**
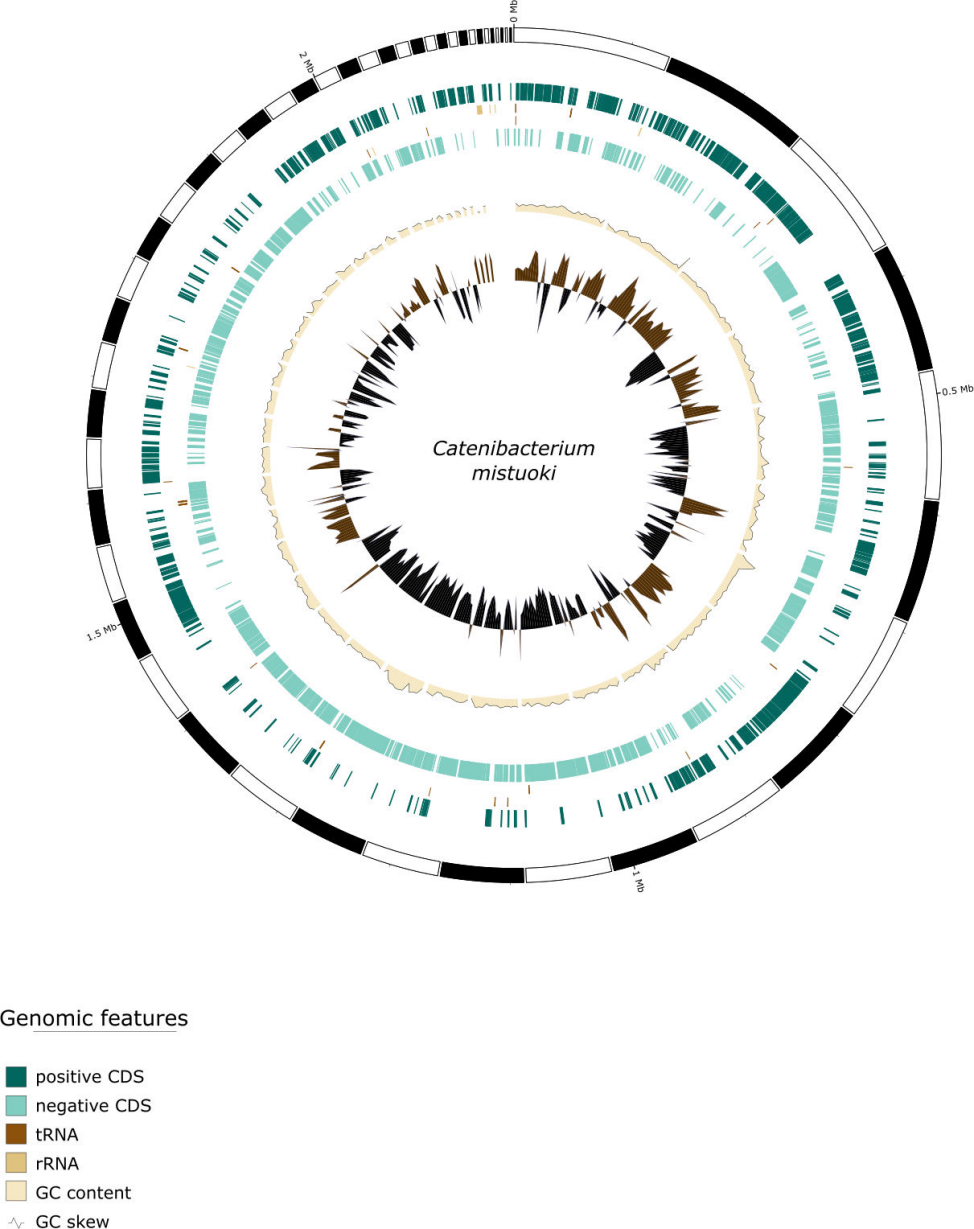
Circular genome representation of *Catenibacterium* sp. generated with GenoVi 0.4.3 showing the GC content, GC skew, and predicted coding sequences across the contigs.

Genome visualization was performed using GenoVi 0.4.3 (11), which generated a circular representation of the assembled genome (Fig. 1). The Genovi plot highlighted features including GC content, GC skew, and predicted coding sequences across the contigs.

## Data Availability

SRA: Nanopore reads: SRR34542285. SRA: Illumina reads: SRR34542286. Biosample: SAMN48894793.
